# Effects of biologically produced gold nanoparticles: toxicity assessment in different rat organs after intraperitoneal injection

**DOI:** 10.1186/s13568-019-0762-0

**Published:** 2019-03-19

**Authors:** Behrooz Yahyaei, Mahnaz Nouri, Sahar Bakherad, Maryam Hassani, Parastoo Pourali

**Affiliations:** 1 Department of Medical Sciences, Shahrood Branch, Islamic Azad University, Shahrood, Iran; 2 Biological Nanoparticles in Medicine Research Center, Shahrood Branch, Islamic Azad University, Shahrood, Iran

**Keywords:** Biologically produced gold nanoparticles, Intraperitoneal injection, Kidney, Liver, Testis

## Abstract

Gold nanoparticles (GNPs) have different usage in the medical field. The plan of the present research was to evaluate the influence of the biologically produced GNPs on some rat organs. GNPs were produced using *Fusarium oxysporum* and their presence was confirmed using spectrophotometer, transmission electron microscope (TEM) and X-ray diffraction (XRD) analyses. The non-toxic and toxic doses of GNPs were determined using MTT assay and were injected intraperitoneally into rats in 3 continuous days and their effects on the kidney, liver and testis were analyzed using microscopic technique. Results revealed that GNPs that were produced had 525 nm absorbance peak and average sizes of about 50 nm, with round and hexagonal shapes. Results from the XRD analysis showed the presence of GNPs in the reaction mixture. MTT assay results revealed that GNPs had somehow toxic effects which depend on their doses. Histological examinations indicated that based on the tested organ, the distribution and effects of GNPs were different which in the testis, the non-toxic dose had no effects and in some parts of the liver and kidney, it induced mild changes. The toxic dose of the GNPs in all the three tested organs induced mild changes. In conclusion, the in vitro and in vivo behaviors of the produced GNPs were different and GNPs even in high concentration induced low changes in the rat organs. This may be due to the short exposure and the use of the biologically produced GNPs.

## Introduction

Gold nanoparticles (GNPs) are known as one of the biocompatible agents and because of their larger surface areas and smaller sizes, they are more biologically active than the bulk gold (Abdelhalim and Moussa 2013). Recently, this nanoparticles is widely used for drug delivery, gene delivery, cancer treatment, biosensors, photothermal therapy, and imaging techniques. Although nanoparticles are known as safe materials, there are different reports about the toxic effects of the used nanoparticles in vivo (Abdelhalim and Moussa 2013; Pourali et al. 2017). Hence, nanotoxicity of the nanosized material is the other approach that should be taken in consideration. There are some available researches which reported that GNPs accumulate in many rat organs (Abdelhalim and Jarrar 2011a, b, c). Size, dose, shape, method of entry and duration of exposure, metabolism, their surface chemistry and immune response to the GNPs are some the factors that affect their toxicity (Zhang et al. 2010). Also, the nanoparticles production type might be an important factor in their toxicity effects. There are three different techniques for nanoparticles production that are named biological, physical and chemical methods (Pourali et al. 2013). The chemical method of nanoparticles production is fast and easy but sometimes existing of some toxic elements on the nanoparticles surfaces and their environmental damages are of its disadvantages. The physical way is time consuming manner and the produced nanoparticles sometimes will not be uniform in their sizes. The biological method of production is named green synthesis which uses microorganisms and plants for nanoparticles production (Pourali et al. 2017). This method of production is environmental friendly and inexpensive. It was reported that some types of fungal and bacterial strains reduce the ions which are imposed to their culture media and by converting them to the nanoparticle forms, the toxicity of the materials will be reduced. This reduction occurred through non-enzymatic and enzymatic ways. In the enzymatic process, the enzymes which are present in or out of the microorganism’s cell act on the toxic ions and reduce them to the nanoparticles and in the non-enzymatic way, some microbial secreted extracellular elements such as the active groups of the proteins and polysaccharides are responsible for bio-production of the nanoparticles (Pourali et al. 2014). Furthermore, another factor is the method of nanoparticles administration which may have impact on their toxicity. It was reported that injection of the nanoparticles through tail vein caused lower toxic effects than those administered through intraperitoneal and oral routes (Zhang et al. 2010). Although there are some available researches about the GNPs production, assessment of their cytotoxicity and administration in vivo (Abdelhalim and Jarrar 2011a, b, c; Abdelhalim and Moussa 2013; Zhang et al. 2010), there is not enough report on the influence of the biologically produced GNPs at their toxic and non-toxic doses in the rat organs. Hence, the recent research tried to produce the GNPs using the fungal strain, *Fusarium oxysporum*. The presence of GNPs was confirmed using different tests and their non-toxic doses were determined using MTT assay. At the final step, the nanoparticles were injected intraperitoneally and their effects on the kidney, liver and testis of the rats were analyzed using microscopic technique.

## Materials and methods

### Fungal strain, culture condition and production of gold nanoparticles

*Fusarium oxysporum* (PTCC 238-21-3) was cultured in sterile Sabouraud dextrose broth (SDB, Merck, Germany) at 27 °C, 150 rpm, for 3 days. The fungal culture medium was centrifuged (5000 rpm for 10 min), and 50*g* of the mycelia was weighed and incubated in ddH2O at 27 °C, 150 rpm, for 3 days. The fungal suspension was centrifuged (5000 rpm for 10 min), then 150 μl of 1 M HAuCl4 (Sigma Aldrich, USA) solution was added to 150 ml of the achieved supernatant to obtain 1 mmol final concentration of HAuCl4 solution. GNPs production was done by incubating the solution at 35 °C, 220 rpm, for 1 day. The control flask containing 150 ml of ddH2O with 150 μl of 1 molar HAuCl4 was incubated under the above condition (Pourali et al. 2017).

### Proving the GNPs formation

Changing of the color of the fungal supernatant from yellow to red, purple, pink or other colors was the first sign of GNPs creation (Pourali et al. 2018).

### Visible spectrophotometer

By the use of Nanodrop spectrophotometer, the absorbance peak of GNPs was monitored. The GNPs had maximum absorbance peak around 510–560 nm which is owing to the surface plasmon resonance (SPR) of the produced nanoparticles, which was one of the signs of nanoparticles production. For this aim, the absorbance spectrum of the fungal supernatant solution was obtained from 350 to 600 nm against the blank solution, SDB (Pourali et al. 2012).

### Transmission electron microscope (TEM)

The exact dimensions and shapes of the obtained GNPs were achieved using Zeiss Leo 910 TEM. For this aim, 20 μl of the GNPs colloidal solution was loaded on a carbon coated grid and after 20 s, the excess of the solution was removed. The dried grid was analyzed under TEM and the pictures were obtained (Yahyaei et al. 2016).

### X-ray diffraction analysis (XRD)

XRD is a technique that can distinguish the GNPs from the other materials in the fungal culture supernatant. The sample was dried and checked under 30 to 80° at 2°θ using Philips automatic X-ray diffractometer (Yahyaei et al. 2018).

### Assessment of the GNPs cytotoxicity in vitro

The produced GNPs were used directly for 3-(4, 5-dimethylthiazol-2-yl)-2, 5-diphenyltetrazolium bromide (MTT) assay. The tested cells were mouse fibroblast cell line NIH3T3 that was obtained from the Pasteur Institute of Iran. The cells (2 × 10^4^) were seeded in a 96 well micro titer plate, each well was loaded by 200 μl of working medium containing Dulbecco’s Modified Eagle’s medium (DMEM), 1% penicillin-streptomycin, and 10% fetal bovine serum (FBS) (Sigma-Aldrich, USA). The plate was incubated in the cell culture incubator for 24 h. In the next day, the surface of the monolayer cells was washed using phosphate buffered saline (PBS) and the wells in each row were loaded by 2 × concentration of 100 μl of the represented medium. The 1st well in one row was loaded by 100 μl of GNPs solution. After mixing, 100 μl of the mixed solution was moved to the second well. This was done for the next well until the 11th well. The last well (i.e. 12th) was the control and included 100 μl of the working medium. The plate was incubated under the above condition and in the next day, the culture medium was removed and 20 μl of the dye solution (5 mg/ml, Sigma-Aldrich, USA) was added to all the wells. The plate was incubated for 2 h in the mentioned condition and the dye was discharged. 100 μl of dimethyl sulfoxide (DMSO, Sigma-Aldrich, USA) was loaded in each well and the plate was incubated in a shaker incubator at 37 °C, 50 rpm, for 20 min. Finally, using ELISA reader spectrophotometer, the absorbance of each well was analyzed at 570 nm. The IC50 of each well was evaluated. For this aim the below formula has been used.

$${\text{IC5}}0 = \left( {{\text{OD}}\;{\text{of}}\;{\text{the}}\; 1 {\text{th}}\;{\text{well}} - {\text{OD}}\;{\text{of}}\;{\text{the}}\; 1 1 {\text{th}}\;{\text{well}}/{\text{OD}}\;{\text{of}}\;{\text{the}}\; 1 1 {\text{th}}\;{\text{well}}} \right) \times 100$$

The first well had the maximum and the 11th well had the minimum concentrations of the GNPs (Pourali et al. 2017).

The toxic and non-toxic doses of the GNPs were achieved from the results of MTT assay. The well before the determined IC50 (which contained higher concentration of the GNPs) was recognized as the toxic and the well after the determined IC50 (which contained lower concentration of the GNPs) was recognized as the non-toxic concentrations of the GNPs. These two concentrations were used in the animal studies.

### Assessment of the GNPs toxicity in vivo

#### Animal studies

Twenty four 10 weeks old Wistar male rats weighing 210–220 g were purchased from Pasteur Institute of Iran about 2 weeks prior to the commencement of the tests. The rats were kept under 12 h light–12 h dark cycles and were given water and food ad libitum. The rats were divided into three separate groups, one group was the control (n = 8) and was administered intraperitoneally with normal saline, another group (n = 8) was administered intraperitoneally with the GNPs in the non-toxic dose and the last group (n = 8) was administered intraperitoneally with the toxic dose of the GNPs for 3 continuous days (Abdelhalim and Moussa 2013; Yahyaei et al. 2016).

#### Histological examination

In day 4, the rats were sacrificed using ketamine and their liver, kidney and testis were collected and weighed. Then the above mentioned organs were fixed using 10% formalin and were embedded in paraffin. After that the sections using microtome apparatus were obtained and were stained using haematoxylin and eosin (H & E) method (Pourali et al. 2016). The variable parameters for the kidney were changes in the glomeruli, Bowman’s capsules, proximal and distal tubules and the presence of hyperemia and inflammation. The variable parameters for the liver were changes in the hepatocytes structure, lobular central vein, portal tract and sinusoidal space. The variable criteria for the testis were changes in the sertoli cells, spermatogenic cells, seminiferous tubules, interstitial tissue and leydig cells. These parameters were compared with each other through one way ANOVA program in SPSS software version 22.

## Results

### Fungal strain, culture condition and production of GNPs

**Fig. 1:**
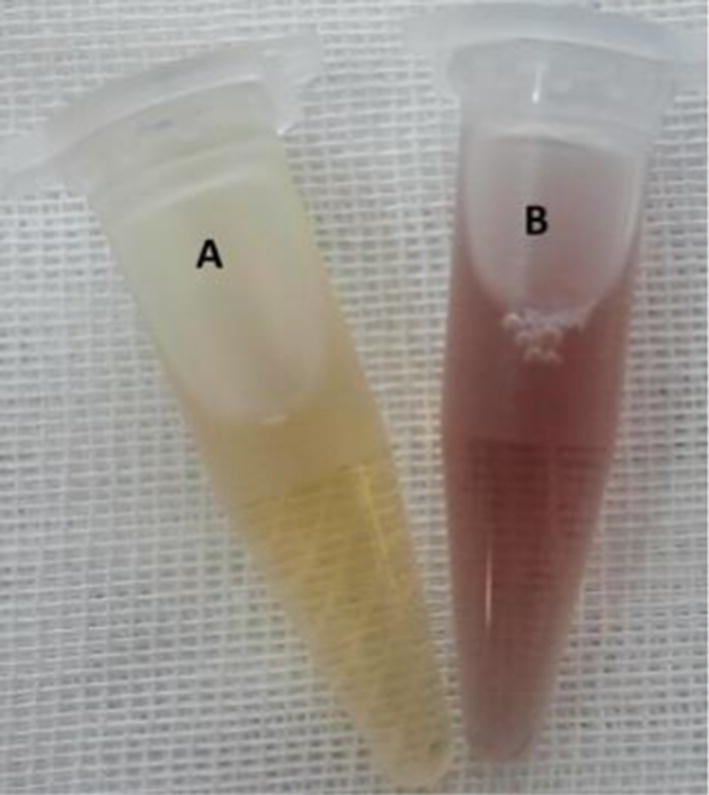
Changes in the color of the fungal extract after biosynthesis of GNPs. A: The fungal extract before and B: after the GNPs production

After the incubation of the *F. oxysporum* extract with 1 mmol final concentration of HAuCl4 solution, the color of the fungal extract changed from light yellow to pink color, which represented the production of GNPs. The color of the control flask did not change (Fig. 1).

### Proving the GNPs formation

#### Visible spectrophotometer

**Fig. 2:**
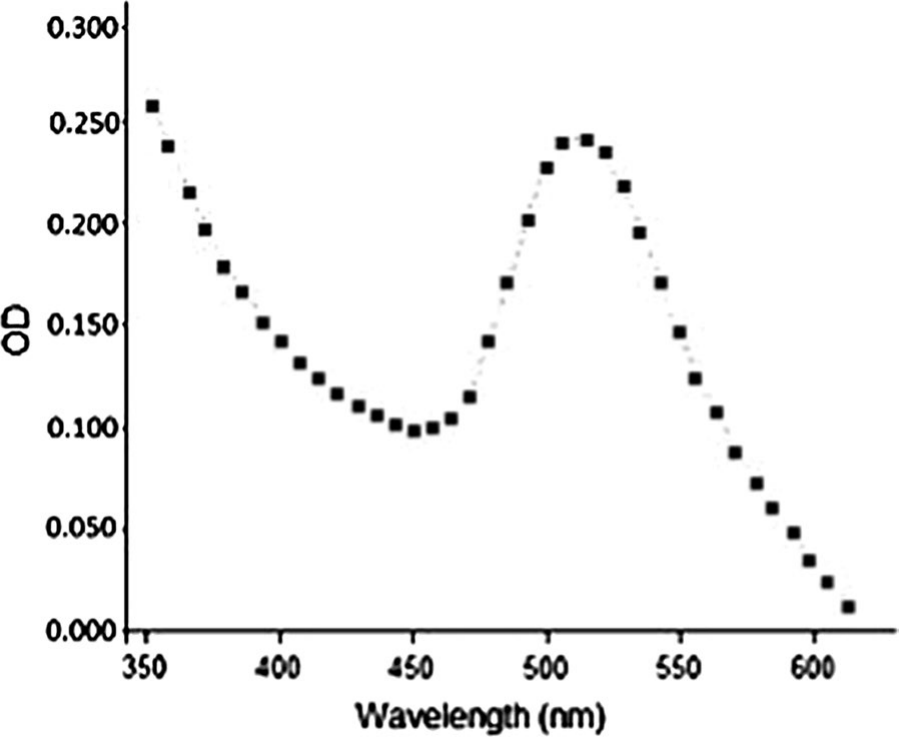
Visible spectrophotometer results of GNPs after 1:5 dilutions with SDB. The maximum absorbance peak around 525 nm is the sign of the presence of GNPs

With the use of Nanodrop spectrophotometer, the absorbance peak of GNPs was monitored from 350 to 600 nm against the blank. Results indicated that GNPs solution had maximum absorbance peak around 525 nm due to the SPR. In order to decrease the turbidity of the mixture, the mixture was diluted 1: 5 using SDB (Fig. 2).

#### Transmission electron microscope (TEM)

**Fig. 3:**
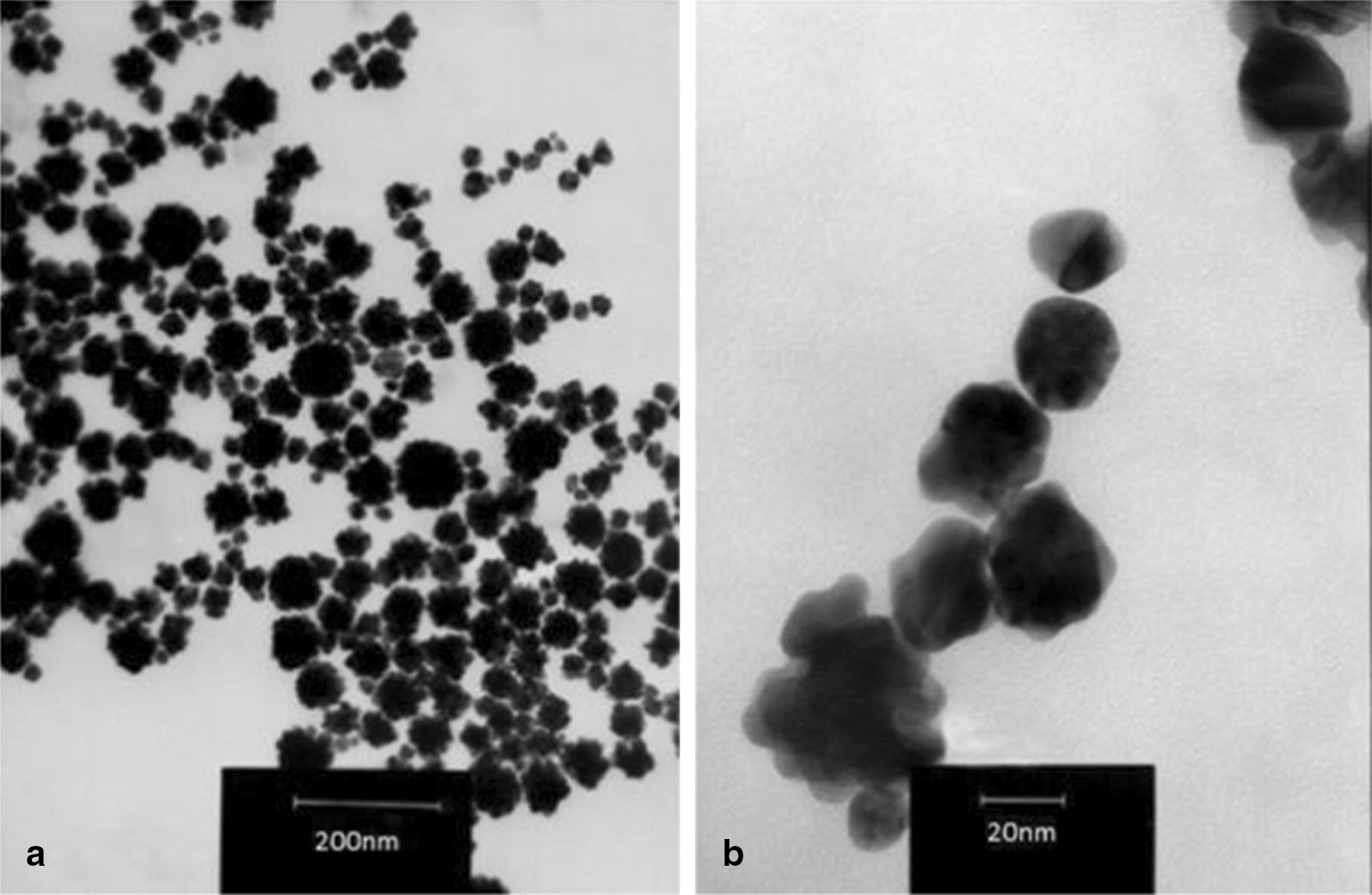
TEM micrographs that were obtained from the biologically produced GNPs. **a** scale bar = 200 nm and **b** scale bar = 20 nm. In both micrographs, the nanoparticles had hexagonal shapes with average sizes of about 50 nm

The obtained GNPs were evaluated through TEM. The average sizes of GNPs were about 50 nm, with hexagonal and round shapes (Fig. 3).

#### X-ray diffraction analysis (XRD)

**Fig. 4:**
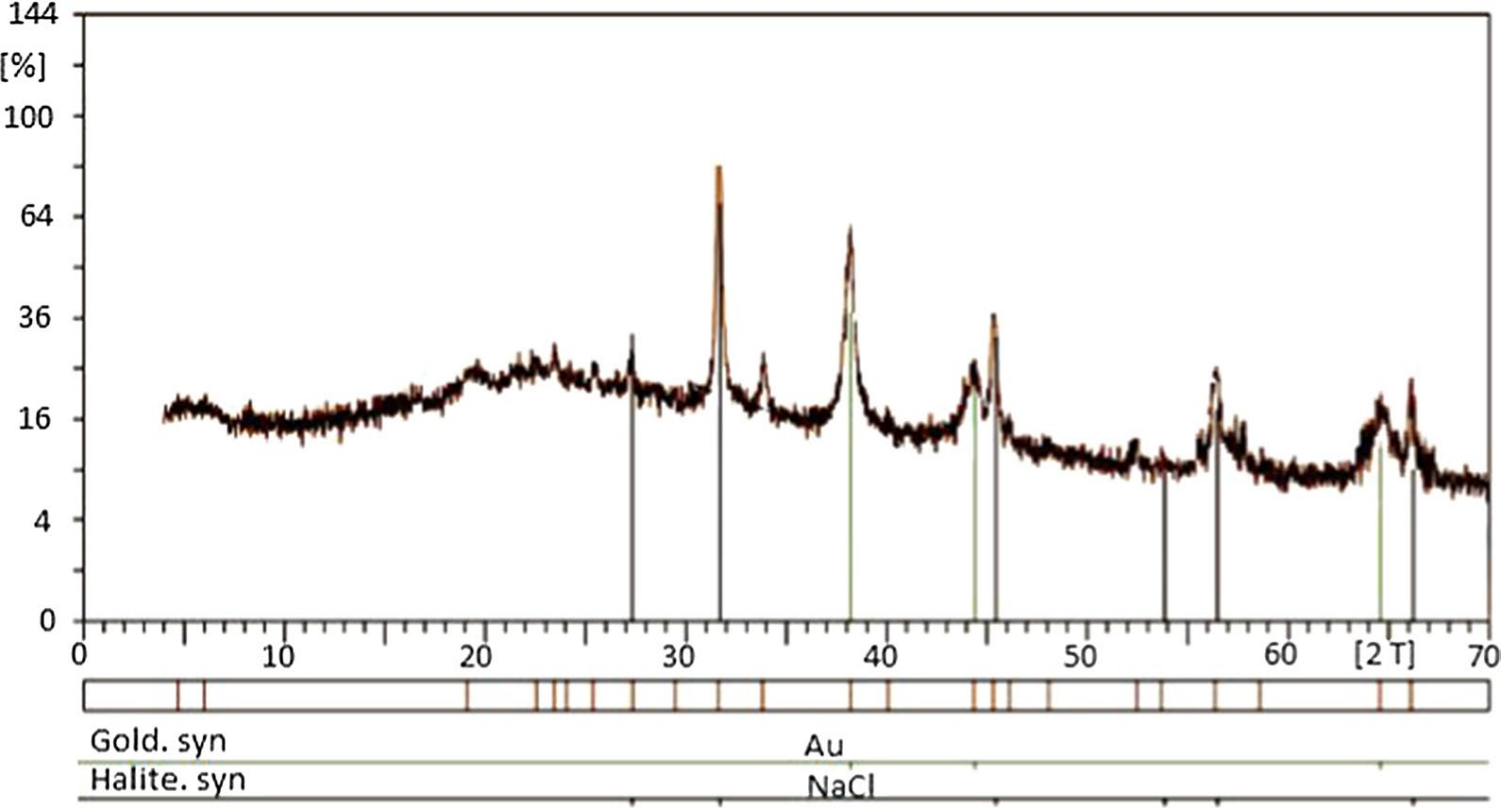
XRD results of the fungal culture supernatant after GNPs production. The presence of impurities in the fungal culture led to the formation of additional peaks

XRD obtained results showed the existence of distinct peaks that belong to the elemental GNPs. The presence of impurities in the fungal culture led to the formation of additional peaks (Fig. 4).

#### Assessment of the GNPs cytotoxicity in vitro

**Fig. 5:**
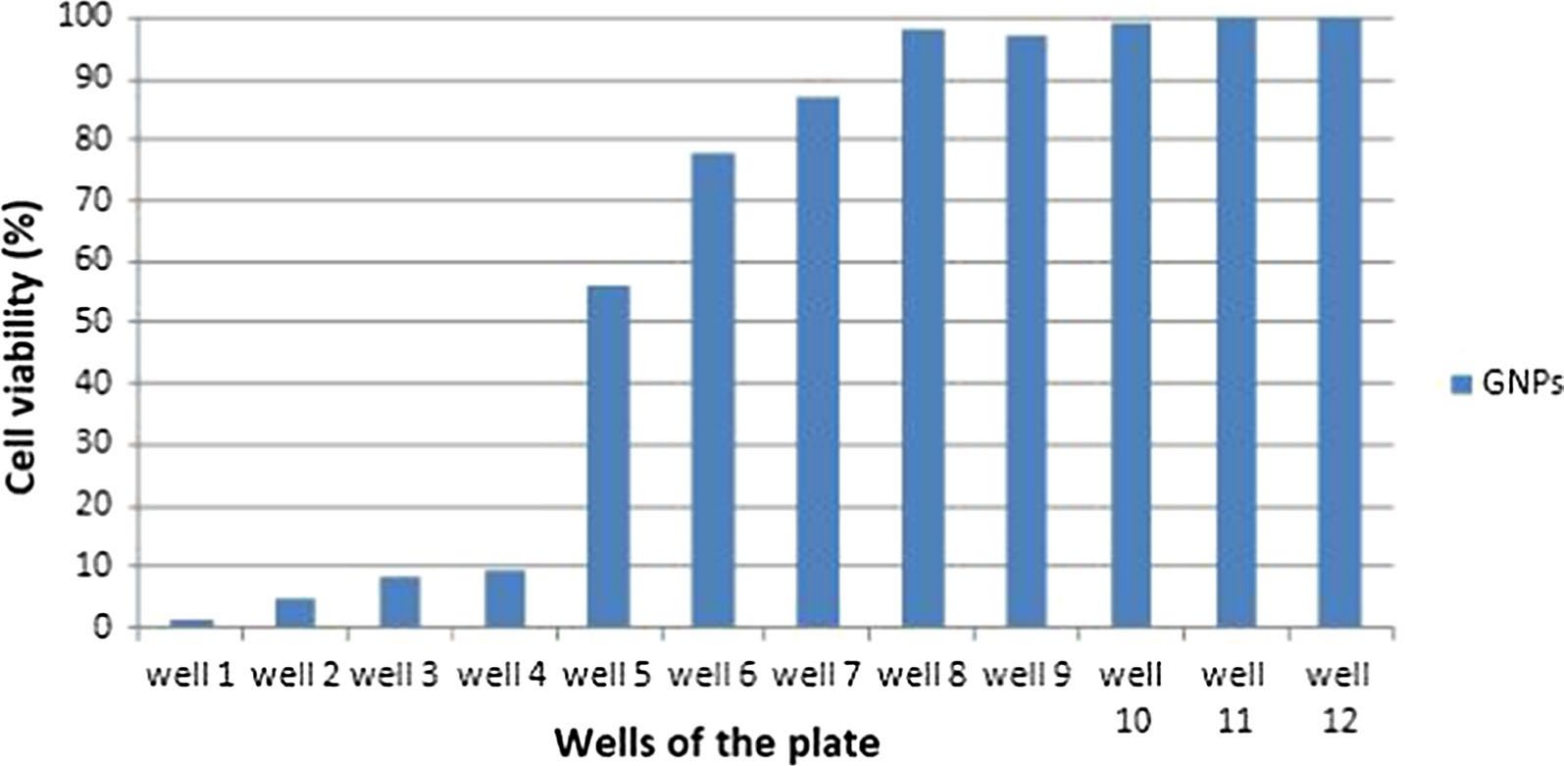
The cell viability percentages obtained from the MTT test. Well 1 contained the maximum and well 11 contained the minimum concentrations of GNPs. Well 12 was the control and had the maximum cell viability

MTT assay results showed that the produced GNPs had toxic effects on mouse fibroblast cell line NIH3T3 which depend on the used doses. The IC50 was determined in the 5th well in which half of the cells survived. This means that the non-toxic dose was in the 6th well and the toxic dose of the GNPs was in the 4th well. Due to the obtained OD values indicated the numbers of the viable cells, the percentages of the viable cells were achieved from the obtained ODs (Fig. 5).

### Assessment of the GNPs toxicity in vivo

#### Animal studies

In order to achieve the non-toxic and toxic doses in the rat model based on the MTT assay results, for toxic dose, the GNPs should be diluted 1/16 and for non-toxic dose, the GNPs should be diluted 1/64 in the rat’s body. Thus for the animal which weighed around 200 g, because approximately 10% of the rat’s body is blood (Donovan and Brown 1995), the animal has 20 ml blood and by using a simple mathematical proportion, the injected toxic and non-toxic doses will be 1.34 and 0.32 ml, respectively. This was done for all the 24 animals. Before the injections, the animals were weighed and the injected doses were achieved. Administration was done for 3 continuous days.

#### Histological examination

**Fig. 6:**
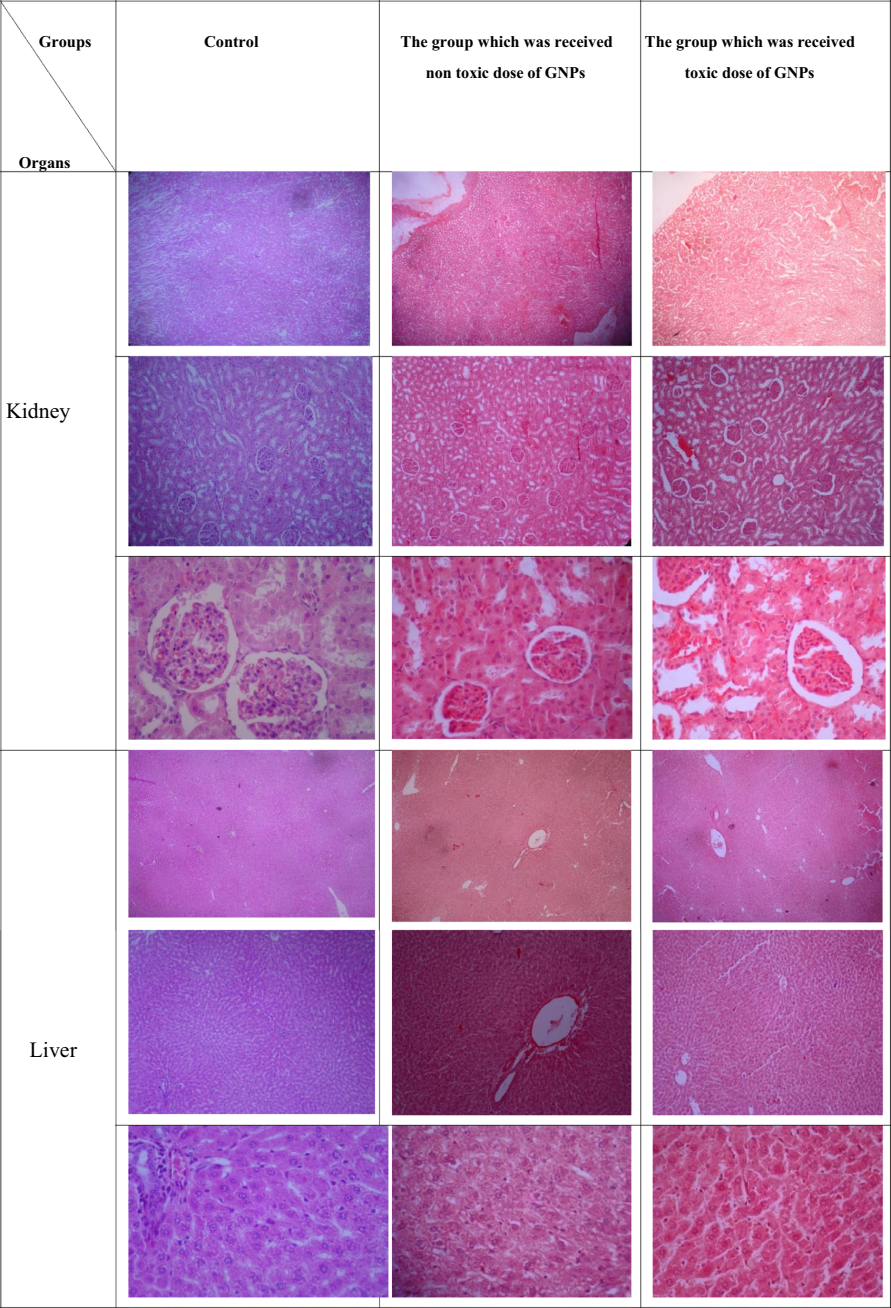
The results of injection of non-toxic and toxic doses of GNPs on the liver and kidney histology in contrast to the control group. The magnification in the first row for each organ is ×40, the second represented ×100 and the third represented ×400 (H&E staining)

**Fig. 7:**
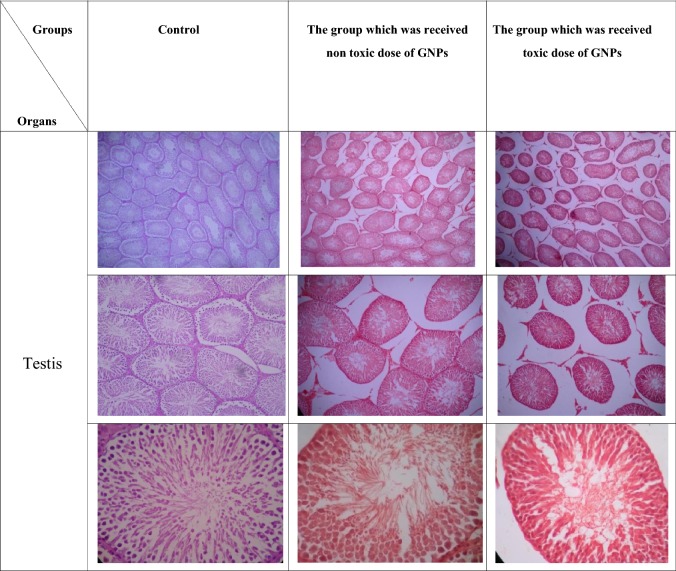
The results of injection of non-toxic and toxic doses of GNPs on the testis histology in contrast to the control group. The magnification in the first row for each organ is ×40, the second represented ×100 and the third represented ×400 (H&E staining)

As it was mentioned earlier, three rat groups were used for this study. The results of the injection of non-toxic and toxic doses of GNPs on the testis, kidney and liver compared to the control group are shown in Figs. 6, 7.

As Figs. 6, 7 represents, in the assessment of the kidney, in the control group, the cortical and central areas of the kidney were completely normal and healthy. In the corneal region, the glomeruli had normal size and number, and the Bowman’s capsules contained natural cells. Urethral had normal size, and distal and proximal tubules showed normal properties. In the central area, renal tubes were healthy and there was no evidence of hyperemia, inflammation, or cysts. In the group which was treated by non-toxic dose of GNPs, the glomeruli placement was good and normal, but small amounts of glomeruli had tendency to shrink and squeeze. Urethral space was well suited and there was no abnormal dilatation. Renal tubes also showed the natural lumen space. The Bowman’s capsule was perfectly shaped, and the mesenchymal cells which made the Bowman’s capsule had normal shape. In the group which was treated by toxic dose of GNPs, in some areas, the numbers of glomeruli were decreased and the urethral space was enlarged and dilated. Some glomeruli shrank with smaller size. Renal tubes showed no particular problem, but there was some hyperemia in their interstitial space. The Bowman’s capsule was completely healthy and its cells had their own natural characteristics.

Based on the ANOVA results, significant difference observed between the control group and the two other groups in the degree of changes in the glomeruli (p value < 0.05), but in the analysis of this parameter, no significant difference observed between the group that was administrated with toxic dose and the one that was treated with non-toxic dose of GNPs (p value > 0.05). In the analysis of the presence of hyperemia and inflammation parameter, there was significant difference between the group that was administered with toxic dose of GNPs and the other two groups (p value < 0.05), but in the analysis of this parameter, no significant difference observed between the control and the group which was administered with non-toxic dose of GNPs (p value > 0.05). In the other criteria, no significant differences observed between all the tested groups (p value > 0.05).

In the analysis of the liver, the hepatocyte cells of the control group represented the normal characteristics with the distinct nuclei. The sinusoidal space, the portal tract area, and the lobular central vein were completely natural. Hepatocyte columns were arranged in a well-defined manner. No collapse in the liver tissue was present. In the samples that were acquired from the group that was administrated with the non-toxic dose of GNPs, hepatocyte columns had a good order. The lobular central vein was dilated in most areas and a slight hyperemia was visible in some specimens in this area. The size and shape of the hepatocyte nucleus were appropriate but some vacuolar changes were observed. The sinusoidal and the portal tract spaces were clearly visible and unchanged. In the samples that were obtained from the group which were administered the toxic dose of GNPs, the hepatocyte cells had a proper structure and only a few of them exhibit slight vacuolar variations. The lobular central vein had a fair distribution and uniformity, and there was no abnormal distension. The sinusoidal space was slightly diffused with hyperemia, which is seen in areas far from the lobular central vein. The Portal tract was unchanged and had normal feature. Based on the ANOVA results, significant difference observed between the control group and the two other groups in the degree of changes in the hepatocytes structure (p value < 0.05), but in the analysis of this parameter, no significant difference observed between the group that was administrated with toxic dose and the one that was treated with non-toxic dose of GNPs (p value > 0.05). In the analysis of changes in the sinusoidal space parameter, there was significant difference between the group that was administered with toxic dose of GNPs and the other two groups (p value < 0.05), but in the analysis of this factor, no significant difference observed between the control and the group that was administrated with non-toxic dose of GNPs (p value > 0.05). In the analysis of changes in the lobular central vein parameter, there was significant difference between the group that was administered with non-toxic dose of GNPs and the other two groups (p value < 0.05), but in the analysis of this parameter, there was no significant difference between the control and the group which was administrated with toxic dose of GNPs (p value > 0.05). In the other examined factors, there no significant differences observed between all the groups (p value > 0.05).

In the assessment of the testis, the samples obtained from the control group had consistent texture, and the diameter and number of seminiferous tubules were suitable and identical. The leydig cells and interstitial tissue had the same size and their count was normal. All the spermatogenic cells and sertoli cells were normal. Also, the amounts of sperms inside the lumen were abundant.

In the group which was treated by-the non-toxic dose of GNPs, no significant changes in the testis tissue were seen. The texture and order of the tissue was quite constant, and in comparison with the control, the only difference was the expansion in the space between the tubes at very small points. The cells of the interstitial tissue had proper sizes and numbers. seminiferous tubules had acceptable counts and sizes. The spermatogenic and sertoli cells did not show any significant changes. Also, the amounts of sperms inside the lumen were abundant.

In the group which was treated by the toxic dose of GNPs, slight structural variations were seen. The distance between the tube and the mediastinal space was increased and a slight amount of hyperemia was seen. Seminiferous tubules had good diameter, but they were less than the control group. Spermatogenic and sertoli cells had normal characteristics, but some internal regions of the seminiferous tubules were empty and free of cells.

Based on the ANOVA results, in the analysis of the presence of changes in the seminiferous tubules and changes in the interstitial tissue and leydig cells parameters, there was significant difference between the group that was administered with toxic dose of GNPs and the other two groups (p value < 0.05), but in the analysis of this parameter, no significant difference observed between the control group and the one which was administered with non-toxic doses of GNPs (p value > 0.05). In the other examined parameters, no significant differences observed between all the tested groups (p value > 0.05).

Also, different factors for the kidney such as changes in the glomeruli, Bowman’s capsules, proximal and distal tubule*s* and the presence of hyperemia and inflammation, for the liver such as changes in the hepatocytes structure, lobular central vein, portal tract and sinusoidal space and for the testis such as changes in the seminiferous tubules, spermatogenic cells, leydig cells, interstitial tissue and sertoli cells, were compared with each other and the degree of the variations was analyzed and shown in Table 1.

**Table 1:** The degree of the changes in different rat organs after been treated with toxic and non-toxic doses of GNPs in the three tested rat groups

Organs and parameters	Groups		
	Control	The group which received non toxic dose of GNPs	The group which received toxic dose of GNPs
Kidney			
Changes in the glomeruli	0	1	1
Changes in the Bowman’s capsules	0	0	0
Changes in the proximal and distal tubules	0	0	0
Present of the hyperemia and inflammation	0	0	1
Liver			
Changes in the hepatocytes structure	0	1	1
Changes in the lobular central vein	0	1	0
Changes in the portal tract	0	0	0
Changes in the sinusoidal space	0	0	1
Testis			
Changes in the seminiferous tubules	0	0	1
Changes in the spermatogenic cells	0	0	0
Changes in the sertoli cells	0	0	0
Changes in the interstitial tissue and leydig cells	0	0	1

In the table, 0 represented no changes, 1 represented mild changes, 2 represented the middle and 3 represented the maximum changes

## Discussion

Studies on nanotoxicity focused on GNPs as a model because they have the highest biocompatibility and the lowest toxicity. Furthermore, they have a high potential for surface modifications and the synthesis of this type of nanoparticles is easy and inexpensive. (Pissuwan et al. 2006).

There are several reports about higher toxicity of the nanoparticles than their bulk materials due to their smaller dimension and larger surface area. Physical dimensions such as surface chemistry, composition, size, shape and the type of the nanoparticles are some important factors which may have impact on their toxicity in vivo. It was reported that nanoparticles cause neurotoxicity, hepatotoxicity, renal toxicity and testis toxicity (Sun et al. 2013).

One factor which influences the toxicity of the nanoparticles is the method of their production. As it was mentioned previously, there are three main types of nanoparticles production which are named chemical, physical and biological techniques. By literature reviewing, it can be distinguished that most of the researches focused on the chemical and physical methods of nanoparticles production and a few papers are available about the in vivo toxic effects of the nanoparticles that are produced by the biological technique.

At the cellular level, it was shown that GNPs uptake occurred through receptor-mediated endocytosis and maximum uptake occurred when the sizes of the used nanoparticles were around 50 nm (Chithrani and Chan 2007). Hence, the size of the nanoparticles influence their cytotoxicity and cellular uptake. Some studies showed that the chemically and physically produced GNPs had low toxic effects in the cell culture. In our previous research, we have used GNPs with sizes of about 50–70 nm as the model of the biologically produced nanoparticles in the cell culture and showed that unlike the technique of production which is known as the safest one, the obtained GNPs had some dose dependent toxicity. But an important factor which must be considered is that the two dimensional cell cultures cannot resemble the complexity of the animal body. For example, in two different researches which analyzed the toxicity of the carbon nanotubes in vitro and in vivo, one report showed that the carbon nanotubes had toxicity in the cell culture and the other showed that the carbon nanotubes had no toxic effects in the animal model (Manna et al. 2005). Parameters such as the used dose, immune response to the nanoparticles, route of exposure in addition to the chemical and physical properties of the nanoparticles may be involved in their toxic effects in vivo (Zhang et al. 2010).

In the present research, we tried to analyze the toxic effects of the GNPs in their determined toxic and non-toxic doses in the animal model which were administered through intraperitoneal injection. This type of administration was chosen on the basis of the previous research which showed that tail vein injection had the lowest and intraperitoneal injection or oral administration of nanoparticles had the highest toxicity in the rat model (Chen et al. 2009; Zhang et al. 2010). We have assayed the differences between the influence of the GNPs that were biologically produced in vitro and in vivo.

There are some information about the elimination of the chemically produced GNPs in the animal blood which is due to their sizes. For example, it was reported that GNPs with the sizes of about 18 nm were eliminated from the blood stream and collected in the spleen and liver (Semmler-Behnke et al. 2008). In another research, it was reported that smaller GNPs (5–15 nm) can easily be distributed in the rat organs in contrast to the bigger ones (50–100 nm) (De Jong et al. 2008). Thus, as GNPs are good candidate for biomedical applications, before any usage of the GNPs with different sizes, shapes and nature for human applications, it is important to evaluate their behavior in the animal model.

In the first step of the examinations, GNPs were produced using *F. oxysporum* that is a well-known organism in the intra and extracellular production of nanoparticles. In the intracellular method of nanoparticles production, the nanoparticles accumulate within the microbial cells where the active components for bio-reduction are placed in the cells, and in the extracellular method of nanoparticles production, the nanoparticles will be produced out of the microbial cells. It was proven that *F. oxysporum* had strong secretion systems and is a good candidate for the extracellular production of nanoparticles. Furthermore, this fungal strain is nonpathogenic for human and its culture is safe, inexpensive and easy. After culturing of the fungal strain, the supernatant of the culture medium was used for extracellular production of GNPs, because the extraction of the nanoparticles is more facile than that of the intracellular technique.

The production of GNPs was confirmed by color changing and the use of spectrophotometer, TEM and XRD. The color of the reaction changed from yellow to pink due to the existence of the GNPs. Based on the shape and size of the GNPs, the obtained color will be altered. Appearance of pink to red color is the sign of the existence of the spherical GNPs in the culture medium (Burda et al. 2005).

The spectrophotometer results revealed that the obtained GNPs showed maximum absorbance peak about 525 nm. This phenomenon was due to the SPR of the GNPs which is the resonant oscillation of conduction electrons after stimulation by light. TEM results indicated that GNPs had sizes of about 50 nm with round and hexagonal shapes. Different magnifications of TEM showed that GNPs had uniform sizes. Finally XRD results revealed the existence of the elemental gold in the reaction mixture. As shown in Fig. 4, there are extra peaks in the XRD spectrum, which are due to the presence of the other materials and impurities in the microbial culture supernatant.

Prior to in vivo analysis, MTT assay was done, and as it was previously shown, the GNPs had toxicity which depended on the used doses. Based on the calculated IC50, the toxic and non-toxic doses of the GNPs were calculated. Because the animal blood will dilute the nanoparticles after injection, thus the nanoparticles were injected in the doses which after dilution in the animal’s body, toxic and non-toxic doses of them would be achieved.

It was reported that after injection of the GNPs, plasma proteins will be adsorbed on the surfaces of the nanoparticles. This may help the opsonisation and accumulation of the nanoparticles which entered into the blood stream. It was shown that the preferred organ in which nanoparticles accumulation occurs is the liver. The accumulation in the other organs is dependent on the type of the nanoparticles, size, shapes and other characteristics (Cardoso et al. 2014). Hence, the recent research tried to find the effects of GNPs on the liver and kidney of the animal model after 3 continuous days of exposure. Our investigation showed that short time exposure to GNPs had low impacts on the liver and kidney. Previous study showed that the short duration exposure to the chemically produced GNPs (50 nm) had minimum effects on the liver enzymes and had no effects on kidney enzymes (Abdelhalim and Moussa 2013) which, agrees with our histological results.

Our study showed that short time exposure to the non-toxic dose of GNPs induced mild changes on hepatocytes and lobular central vein and toxic dose of GNPs induced mild changes on hepatocytes and sinusoidal space which in long term exposure may cause infiltration and disability of bile and blood transferring and finally liver dysfunction and degeneration process. Furthermore, these two different doses had the same effects (i.e. mild changes) on the glomeruli of the kidney which in long term exposure may cause low filtration rate and kidney dysfunction. Overall, it is important to note that the changes in some parts of the mentioned organs are classified to the mild changes and this data again shows that the results that were obtained from in vivo studies are different from the in vitro ones.

Another organ that was analyzed was the testis. Testis was chosen as the organ in which the blood-testis barrier acts as the filter. In mammalians, an example of one of the tightest junction is blood-testis barrier which is made from the sertoli cells. The purpose of the presence of this barrier is to protect the meiosis process from any harmful agents that may exist in the animal’s blood (Mruk and Cheng 2015). Therefore, we tried to evaluate the toxic effects and penetration of the GNPs in this organ as well. This barrier acts like the blood–brain barrier which guards the brain from unfavourable materials.

Previous research demonstrated that small sizes of GNPs could penetrate the blood–brain barrier, and by the use of the ion channel blockers, this penetration can be under control. They showed that there were no adverse effects on the animals’ brain and suggested the use of the GNPs for drug delivery aims (Hainfeld et al. 2013). Although it was shown that nanoparticles have less effects on the blood–brain barrier, Prakash et al. showed that silver nanoparticles (SNPs) with sizes around 20–100 nm could penetrate blood–brain and blood-testis barriers and induce impairments in the function of the central nervous system (CNS) and teratogenic outcomes in the fetus of the animals which were treated by SNPs (Prakash et al. 2018).

The present study showed that the blood-testis barrier has some effects in the penetration of the GNPs to the testis. GNPs had no toxicity on the sertoli cells that are involved in the production of blood-testis barrier but by the administration of the toxic dose of GNPs, they had mild changes on the seminiferous tubules, which in long term exposure may have impact on the meiosis and therefore act on the spermatozoa. Moreover, toxic dose of GNPs administration had mild changes on leydig cells which in long term exposure may affect the production of testosterone.

Recent research showed that the produced GNPs even in their toxic doses had mild changes in different organs of the rat. This may be due to the use of the biologically produced GNPs, their chemistry, shapes and sizes. This research tried to analyze the toxicity of two different doses of the GNPs on the liver, kidney and testis of the rat model. Analysis of the three other important organs, heart, spleen and brain, are under research and in future we will publish the results.

The aim of this research was to evaluate the toxic effects and distribution of the 50–70 nm GNPs in the animal organs after 3 continuous days of administration. Results from the present study showed that the in vitro and in vivo behaviors of the GNPs are different. Overall, our results showed that the GNPs have easy access to the blood and all the three tested organs but firstly, the non-toxic dose of GNPs had little effects on the tested organs and in the case of the testis, it imposes no changes. Secondly, if administrations of the toxic and non-toxic doses of GNPs had effects, their effects were somewhat similar to each other in the tested organs. Thirdly, the used route of administration is known as the most toxic way of entrance of GNPs to the animal model, which should be compared with the other routes of administration in future. Furthermore, it is recommended to compare the toxic effects of the GNPs that will be produced by the chemical and physical techniques with the biological ones.

## References

[CR1] Abdelhalim MAK, Jarrar BM (2011). Gold nanoparticles administration induced prominent inflammatory, central vein intima disruption, fatty change and Kupffer cells hyperplasia. Lipids Health Dis.

[CR2] Abdelhalim MAK, Jarrar BM (2011). Gold nanoparticles induced cloudy swelling to hydropic degeneration, cytoplasmic hyaline vacuolation, polymorphism, binucleation, karyopyknosis, karyolysis, karyorrhexis and necrosis in the liver. Lipids Health Dis.

[CR3] Abdelhalim MAK, Jarrar BM (2011). Renal tissue alterations were size-dependent with smaller ones induced more effects and related with time exposure of gold nanoparticles. Lipids Health Dis.

[CR4] Abdelhalim MAK, Moussa SAA (2013). The gold nanoparticle size and exposure duration effect on the liver and kidney function of rats: in vivo. Saudi J Biol Sci.

[CR5] Burda C, Chen X, Narayanan R, El-Sayed MA (2005). Chemistry and properties of nanocrystals of different shapes. Chem Rev.

[CR6] Cardoso E, Londero E, Ferreira GK, Rezin GT, Zanoni ET, de Souza Notoya F, Leffa DD, Damiani AP, Daumann F, Rohr P (2014). Gold nanoparticles induce DNA damage in the blood and liver of rats. J Nanopart Res.

[CR7] Chen Y-S, Hung Y-C, Liau I, Huang GS (2009). Assessment of the in vivo toxicity of gold nanoparticles. Nanoscale Res Lett.

[CR8] Chithrani BD, Chan WC (2007). Elucidating the mechanism of cellular uptake and removal of protein-coated gold nanoparticles of different sizes and shapes. Nano Lett.

[CR9] De Jong WH, Hagens WI, Krystek P, Burger MC, Sips AJ, Geertsma RE (2008). Particle size-dependent organ distribution of gold nanoparticles after intravenous administration. Biomaterials.

[CR10] Donovan J, Brown P (1995). Blood collection. Curr Protoc Immunol.

[CR11] Hainfeld JF, Smilowitz HM, O’connor MJ, Dilmanian FA, Slatkin DN (2013). Gold nanoparticle imaging and radiotherapy of brain tumors in mice. Nanomedicine.

[CR12] Manna SK, Sarkar S, Barr J, Wise K, Barrera EV, Jejelowo O, Rice-Ficht AC, Ramesh GT (2005). Single-walled carbon nanotube induces oxidative stress and activates nuclear transcription factor-κB in human keratinocytes. Nano Lett.

[CR13] Mruk DD, Cheng CY (2015). The mammalian blood-testis barrier: its biology and regulation. Endocr Rev.

[CR14] Pissuwan D, Valenzuela SM, Cortie MB (2006). Therapeutic possibilities of plasmonically heated gold nanoparticles. Trends Biotechnol.

[CR15] Pourali P, Baserisalehi M, Afsharnezhad S, Behravan J, Alavi H, Hosseini A (2012). Biological synthesis of silver and gold nanoparticles by bacteria in different temperatures (37 C and 50 C). J Pure Appl Microbiol.

[CR16] Pourali P, Baserisalehi M, Afsharnezhad S, Behravan J, Ganjali R, Bahador N, Arabzadeh S (2013). The effect of temperature on antibacterial activity of biosynthesized silver nanoparticles. Biometals.

[CR17] Pourali P, Yahyaei B, Ajoudanifar H, Taheri R, Alavi H, Hoseini A (2014). Impregnation of the bacterial cellulose membrane with biologically produced silver nanoparticles. Curr Microbiol.

[CR18] Pourali P, Razavian Zadeh N, Yahyaei B (2016). Silver nanoparticles production by two soil isolated bacteria, *Bacillus thuringiensis* and *Enterobacter cloacae*, and assessment of their cytotoxicity and wound healing effect in rats. Wound Repair Regenerat.

[CR19] Pourali P, Badiee SH, Manafi S, Noorani T, Rezaei A, Yahyaei B (2017). Biosynthesis of gold nanoparticles by two bacterial and fungal strains, *Bacillus cereus* and *Fusarium oxysporum*, and assessment and comparison of their nanotoxicity in vitro by direct and indirect assays. Electron J Biotechnol.

[CR20] Pourali P, Yahyaei B, Afsharnezhad S (2018). Bio-synthesis of gold nanoparticles by *Fusarium oxysporum* and assessment of their conjugation possibility with two types of β-lactam antibiotics without any additional linkers. Microbiology.

[CR21] Prakash P, Royana S, Sankarsan P (2018). Multi-organ teratogenesis sequels of bigger size particles colloidal silver in primate vertebrates. J Cytol Histol.

[CR22] Semmler-Behnke M, Kreyling WG, Lipka J, Fertsch S, Wenk A, Takenaka S, Schmid G, Brandau W (2008). Biodistribution of 1.4-and 18-nm gold particles in rats. Small.

[CR23] Sun J, Zhang Q, Wang Z, Yan B (2013). Effects of nanotoxicity on female reproductivity and fetal development in animal models. Int J Mol Sci.

[CR24] Yahyaei B, Peyvandi N, Akbari H, Arabzadeh S, Afsharnezhad S, Ajoudanifar H, Pourali P (2016). Production, assessment, and impregnation of hyaluronic acid with silver nanoparticles that were produced by *Streptococcus pyogenes* for tissue engineering applications. Appl Biol Chem.

[CR25] Yahyaei B, Manafi S, Fahimi B, Arabzadeh S, Pourali P (2018). Production of electrospun polyvinyl alcohol/microbial synthesized silver nanoparticles scaffold for the treatment of fungating wounds. Appl Nanosci.

[CR26] Zhang X-D, Wu H-Y, Wu D, Wang Y-Y, Chang J-H, Zhai Z-B, Meng A-M, Liu P-X, Zhang L-A, Fan F-Y (2010). Toxicologic effects of gold nanoparticles in vivo by different administration routes. Int J Nanomed.

